# Green Synthesis of Furfural and Hydroxymethylfurfural from Various Substrates Using SupraDES under Microwave Irradiation: Techno-Economic Evaluation

**DOI:** 10.1021/acsomega.5c01297

**Published:** 2025-06-04

**Authors:** Lorena Cristina de Andrade Leles, Gabriel Abranches Dias Castro, Jaderson Lopes Milagres, Juliana Ribeiro Paes, Leonarde Nascimento Rodrigues, Ricardo de Carvalho Bittencourt, Marcelo Moreira da Costa, Sergio Antonio Fernandes

**Affiliations:** a Grupo de Química Supramolecular e Biomimética (GQSB), Departamento de Química, 28120 Universidade Federal de Viçosa, Viçosa, MG 36570-900, Brazil; b Departamento de Ciências Exatas e da Terra, 153971 Universidade do Estado de Minas Gerais, Ubá, MG 36500-000, Brazil; c Departamento de Física, Centro de Ciências Exatas, 28120 Universidade Federal de Viçosa, Viçosa, MG 36570-900, Brazil; d Departamento de Engenharia Florestal, 28120 Universidade Federal de Viçosa, Viçosa, MG 36570-900, Brazil

## Abstract

The urgent need to replace fossil resources with renewable materials has driven the development of technologies to convert lignocellulosic biomass and its derived monosaccharides into valuable chemicals such as furfural (FF), 5-hydroxymethylfurfural (HMF), and levulinic acid (LA). This study employs a green and renewable solvent composed of 2-hydroxypropyl-β*-*cyclodextrin (HP-β-CD) and LA, a supramolecular deep eutectic solvent (SupraDES), to synthesize FF from d-xylose. Microwave irradiation (MI) and *p*-sulfonic acid calix[4]­arene (CX4SO_3_H) were used as the heating method and organocatalyst, respectively. The SupraDES was characterized by using Raman spectroscopy, infrared spectroscopy, and thermogravimetric analysis. The results demonstrate the potential of this environmentally friendly system for efficient conversion of monosaccharides derived from lignocellulosic biomass, achieving a maximum yield of 70.6% of FF from d-xylose and 29.7% HMF from Dahlia tuber inulin, using heating via MI at 150 °C for 10 min, 150 mg of SupraDES, 3.0 mL of butyl acetate, and 1.0 mol % CX4SO_3_H. The results of the techno-economic assessment of FF production showed highly positive financial outcomes, with strong economic viability and a quick capital payback.

## Graphical abstract

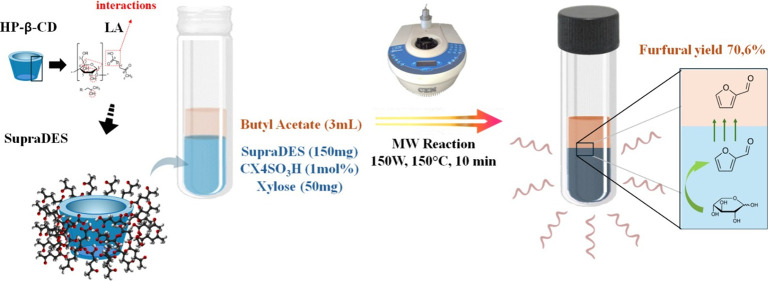

## 1 Introduction

Replacing fossil resources with raw materials from renewable sources has sparked increasing concern in developing new technologies and adapting existing ones to convert renewable materials into valuable products for society, such as fuels, energy, and chemical inputs.^1–3^ An example of this is the conversion of lignocellulosic biomasses, rich in polysaccharides, into high-value-added chemical products like furfural (FF), 5-hydroxymethylfurfural (HMF), and levulinic acid (LA).^4–7^ The necessity to develop new technologies arises due to the highly complex chemical matrix of lignocellulosic biomass, which presents numerous challenges for its viability as a substitute for fossil resources.^8^

Therefore, various technological systems are under development to address this burgeoning demand. A notable example is the utilization of Quaker Oats’ batch technology for FF production, pioneering its industrial-scale application in this specific chemical platform. In this process, oat husks, when mixed with sulfuric acid (2% *m*/*m*), undergo heating at 153 °C for 5 h, resulting in FF yields of approximately 50%. However, an intrinsic limitation of this approach, extending to numerous other methodologies for lignocellulosic biomass and its derivative conversion, lies in the utilization of mineral acids like sulfuric and phosphoric acids as catalysts. Apart from their potential human toxicity and environmental impact, these acids exhibit corrosive characteristics, thereby compromising the overall process performance and leading to reactor deterioration.^3,4^

Therefore, many current researches invest their efforts in the search for efficient, environmentally friendly, viable systems, and less aggressive catalysts for the synthesis of FF and other platform molecules.^4,6,9–11^ Various approaches have been explored in this regard, including the use of organocatalysts,^12^ natural catalysts,^13^ monophasic and biphasic solvent systems,^10,14–17^ and ionic liquids,^18^ among others. In this context, several studies have demonstrated promising results with the use of calix[4]­arene organocatalysts and deep eutectic solvents (DES) in biomass valorization processes.^14–17,19,20^

Calix­[*n*]­arenes offer significant advantages, including mild reaction conditions, high selectivity, and the ability to be easily modified for specific reactions.^19,21^ Their acidic or basic properties make them effective in various catalytic processes.^22^ Compared to conventional acids, calix­[*n*]­arenes are more environmentally friendly due to their low toxicity and recyclability, which reduces the need for frequent catalyst replacement.^1,23^ Their use can enhance biomass conversion efficiency while promoting more sustainable industrial practices with reduced environmental and economic impacts.

The DES are characterized as a combination of two or more substances—one acting as a hydrogen bond acceptor (HBA) and the other as a donor (HBD)—exhibiting, at certain molar ratios, a lower melting temperature than the pure components.^24,25^ They offer notable advantages that justify their application in biorefinery processes, such as low vapor pressure, adjustable viscosities, nonflammability, ease of preparation, and the ability to be produced from natural, recyclable, and nontoxic substances, including sugars, organic acids, and their salts.^26,27^

In this context, many studies report the use of deep eutectic solvents (DES) for the production of furanics compounds.^19,28–30^ Sun et al.^3^ proposed a biphasic system composed of a water-soluble DES and cyclopentyl methyl ether for the pretreatment of eucalyptus aimed at FF production, achieving yields of up to 80%. Appiah et al.^31^ investigated the catalytic conversion of xylose into FF using DESs formulated with choline chloride (ChCl) and malic acid, in combination with catalysts such as ferric chloride and sodium chloride, reaching yields of up to 87% FF from xylose. Delgadillo et al.^29^ employed a biphasic system consisting of a ChCl-based DES, water, *p*-toluenesulfonic acid (PTSA), xylose, and ethyl acetate, achieving a furfural yield of 50%.

A less explored class is supramolecular deep eutectic solvents (SupraDES), formed by substances acting as supramolecules, such as calix­[*n*]­arenes, cyclodextrins, and cucurbit­[*n*]­urils, which can enhance DES properties in terms of functionality.^32–37^ Although the scientific literature on SupraDES and its applications is currently limited due to its recent introduction, the existing data indicate that β-cyclodextrins (β-CDs) are the most commonly used.^38,39^ This is likely due to their cost-effectiveness and the optimal size of the CD cavity, which suits many applications well,^40^ such as extraction and solubilization of essential oils,^27^ drug solubilization,^36^ extraction of phytochemicals,^41^ polymer synthesis,^42^ desulfurization,^43^ and extraction of phenolic compounds,^44^ among others. The oligomeric cyclic structure of cyclodextrin glycopyranoses exposes hydroxyl groups capable of forming hydrogen bonds, which allows these compounds to form a eutectic mixture with hydrogen bond acceptors and/or donors. Supramolecular host–guest interactions are of great interest as they can stabilize reaction intermediates, leading to higher yields and selectivity.^15,45^ Therefore, combining cyclodextrins and natural acids represents an attractive and still underexplored alternative for converting carbohydrates into platform molecules.^36,46,47^ A promising candidate is levulinic acid (LA), derived from biomass, which offers significant advantages, including its renewable origin and versatility in producing high-value-added chemicals.^48,49^

Therefore, in this work, the objective was to employ a SupraDES consisting of 2-hydroxypropyl-β-cyclodextrin (HP-β-CD) and LA for the conversion xylose for FF and HMF. For this, heating via microwave irradiation (MI) and *p*-sulfonic acid calix[4]­arene (CX4SO_3_H) were used as an organocatalyst. The choice for this form of heating and organocatalyst was made because both have already been reported to be highly efficient in these chemical processes.^14,15,19^

## 2 Experimental Section

### 2.1 Materials and Instruments

All reagents and solvents were purchased with a high degree of purity and recommended by the manufacturers for synthesis and/or analysis. Corn cob biomass was acquired in Ervália (Minas Gerais, Brazil) characterized for moisture content, ash content, extractive content, soluble and insoluble lignin, and carbohydrate content.^19^ The corn cob composition is 9.18 ± 0.15% moisture, 2.84 ± 0.87% ash, 5.67 ± 0.07% extractives, 2.95 ± 0.02% soluble lignin, 15.06 ± 0.05% insoluble lignin, 34.2 ± 0.3% glucose, 1.8 ± 0.0% galactose, 3.9 ± 0.1% arabinose, and 25.5 ± 0.1% d-xylose. The experiments were assisted in an MI, employing a CEM discovery microwave reactor and 10 mL Pyrex glass sealed tubes. The temperature of the experiments was monitored using an internal probe. The quantification and identification of FF and HMF were carried out using gas chromatography coupled with mass spectrometry (GCMS) on a SHIMADZU GCMS-QP2010C Ultra. The spectra of the obtained products were compared with the spectra of commercially available FF and HMF standards to confirm the identity of the compounds. Infrared (IR) spectra were obtained using an Agilent Cary 630 FTIR. In the Raman scattering analyses, the Renishaw InVia micro-Raman system was utilized. Thermogravimetric analysis was performed on a PerkinElmer simultaneous thermal analyzer (STA) 6000.

### 2.2 Synthesis of CX4SO_3_H

The CX4SO_3_H was synthesized and characterized in the laboratory according to a previously reported methodology^15,50–52^

### 2.3 SupraDES Preparation Procedure

The SupraDESs were prepared by adapting a methodology previously reported.^27,36^ This involved mixing HP-β-CD and LA in a molar ratio of 1:32, HP-β-CD and citric acid (CA) in a molar ratio of 1:14, HP-β-CD and lactic acid (LaA) in a molar ratio of 1:35, and HP-β-CD and acetic acid (AA) in a molar ratio of 1:32. The mixtures were then heated to 80 °C with stirring until a clear liquid was obtained, and no solid particles were visible, typically taking around 10 min. Subsequently, the SupraDESs were transferred to a desiccator and left to cool until they reached room temperature.

### 2.4 Characterization of the SupraDES

The SupraDES utilized in this study, consisting of HP-β-CD and LA, underwent characterization through Raman spectroscopy, thermogravimetric analysis, and infrared (IR) spectroscopy. To conduct the Raman analyses employed an argon laser (785 nm), helium–neon laser (633 nm), and a 50x objective lens (NA = 0.75), resulting in a focal spot diameter of approximately 1 μm. It is a single monochromator system with a spectral resolution of 1 cm. In the thermogravimetric analysis, the sample masses ranged from 10.0 to 50.0 mg, with a heating rate of 10 °C min^–1^ under a nitrogen flow. Temperature data from the thermograms were recorded at intervals of 0.1 °C over a range of 30–900 °C. The IR spectra were recorded in the wavenumber range from 4000 to 650 cm^–1^.

### 2.5 General Procedure for the Synthesis of FF and HMF

For the synthesis of FF from d-xylose, a mass ratio of 1:3 of carbohydrate (50.0 mg) to SupraDES (150.0 mg), 1.0 mol % CX4SO_3_H (2.3 mg), and 3.0 mL of butyl acetate formed a biphasic system. This mixture was added to a Pyrex glass tube, which, after being sealed, was taken to the MW reactor cavity and heated to 150 °C for 10 min, using a maximum power of 150 W. After completion of the reaction, the organic phase was separated from the SupraDES. For FF quantification, this mixture was transferred to a 5.0 mL volumetric flask, which had its volume adjusted with ethyl acetate.

For the synthesis of HMF from d-fructose or inulin, a 1:3 mass ratio of carbohydrate (60.0 mg) to SupraDES (150.0 mg), 1.0 mol % CX4SO_3_H (2.3 mg), and 3.0 mL of butyl acetate were mixed. This mixture was added to a Pyrex glass tube, which, after being sealed, was taken to the cavity of the MW reactor and heated to 150 °C for 10 min, using a maximum power of 150 W. After completion of the reaction, the organic phase was added was separated from SupraDES. To quantify HMF, this mixture was transferred to a 5.0 mL volumetric flask, which had its volume adjusted with ethyl acetate.

### 2.6 Procedure for Sample Preparation and Quantification of FF and HMF

To quantify FF and HMF in each sample, we adopted the methodology previously outlined by Castro and Fernandes.^17^ First, the organic phase was filtered, and the resulting solution was transferred to a 5 mL volumetric flask, which was then filled to the mark with ethyl acetate. A 312 μL aliquot was taken from this volumetric flask and transferred to a 1.0 mL vial. To this vial was added 688 μL of a 1.45 mg/mL solution of 1,3,5-trimethoxybenzene (TMB, internal standard), resulting in a final concentration of 1.00 mg/mL of TMB. Subsequently, the samples underwent analysis via GCMS, employing a method characterized by the following specifications: SBP-5 column, 30 m in length, with an inner diameter of 0.25 mm; helium employed as the carrier gas; injector temperature set at 290 °C; initial oven temperature set at 40 °C (maintained for 3 min), followed by a ramp of 10 °C min^–1^ up to 100 °C, and then a rapid increase of 60 °C min^–1^ to 250 °C (held for 0.5 min); and injection volume of 1 μL. FF and HMF quantification was carried out by utilizing an external standard method, with TMB serving as the internal standard. Standard solutions, prepared in ethyl acetate, contained FF and HMF at concentrations ranging from 0.10 to 1.50 mg mL^–1^, while TMB maintained a constant concentration of 1.00 mg mL^–1^ (Figures S1–S3). Injection of 1 μL of standard solutions into the GCMS system was performed. Calibration curves (*R* ^2^ = 0.998) were generated by plotting the FF area against the TMB area for each standard injection. The FF concentration was determined based on this calibration curve, and its yield was calculated using eq 1, with d-xylose or l-arabinose serving as the initial substrate, where *n*_FF_ represents the number of moles of FF formed and *n* d‑xylose or l‑arabinose represents the number of moles of d-xylose or l-arabinose:

$$\mathrm{FF}\left(\%\right)=\frac{n_{\mathrm{FF}}}{n_{D‐\mathrm{xylose}\;\mathrm{or}\;L‐\mathrm{arabinose}}}\times 100$$ 1

The HMF concentration was also determined based on this calibration curve, and its yield was calculated using eq 2, with d-fructose or inulin serving as the initial substrate, where *n*_HMF_ represents the number of moles of HMF formed and *n* d‑fructose represents the number of moles of d-fructose or inulin:

$$\mathrm{HMF}\left(\%\right)=\frac{n_{\mathrm{HMF}}}{n_{D‐\mathrm{fructose}\;\mathrm{or}\;\mathrm{inulin}}}\times 100$$ 2

### 2.7 Techno-Economic Assessment (TEA) Methodology

A techno-economic assessment was conducted for the production of FF from d-xylose using the SupraDES/CX4SO_3_H/butyl acetate system. The process began with the hydrolysis of bamboo to extract d-xylose, followed by its conversion into FF.

To estimate operational expenditures (OPEX), specific costs for each input were calculated based on industry pricing. Capital expenditures (CAPEX) included the installation of hydrolysis and catalytic conversion reactors (Table 1), as well as evaporation and purification systems. Financial metrics such as the internal rate of retorne (IRR), payback period, net present value (NPV), and return on investment (ROI) were analyzed to evaluate the economic feasibility

**Table 1:** Capital Expenditure (CAPEX) Breakdown for the Furfural Production Process, Including Costs for Hydrolysis, Conversion, and Purification Equipment^a^

category	value
hydrolysis reactor (USD)	60,000
d-xylose to FF conversion reactor (USD)	75,000
evaporation and purification (USD)	90,000
total CAPEX (USD)	225,000

a The capital expenditure (CAPEX) estimates were determined based on the authors’ technical expertise and informed engineering judgment, reflecting realistic assumptions for each processing unit.

## 3 Results and Discussion

### 3.1 Characterization of the SupraDES

In Figure 1I, the infrared spectra of HP-β-CD, LA, and SupraDES are shown in the regions from 4000 to 650 cm^–1^. Figure 1I illustrates that the band in the region from 3600 to 3000 cm^–1^, corresponding to the O–H stretching, is shifted to 3431 cm^–1^ in SupraDES compared to HP-β-CD (3330 cm^–1^) and LA (3100 cm^–1^). This shift occurs due to an interaction between HP-β-CD and the −OH of the carboxyl group in LA. Additionally, there is an increase in the intensity of the C–H band at 2974 cm^–1^ in SupraDES, indicating a hydrophobic interaction between the respective components.

**Figure 1:**
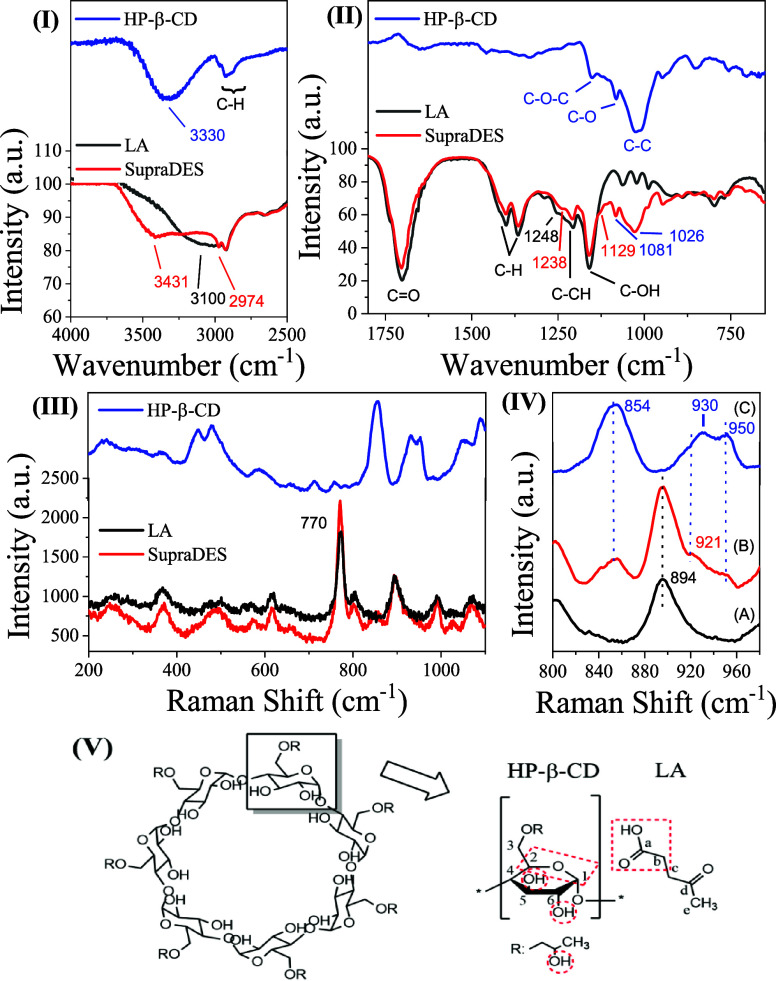
Spectra obtained for SupraDES, LA, and HP-β-CD. (I) Infrared spectrum in the region from 4000 to 2500 cm^–1^. (II) Infrared spectrum in the region from 1800 to 650 cm^–1^. (III) Raman spectrum. (IV) Raman spectrum in the region from 800 to 980 cm^–1^. (V) Illustrative image showing the interaction in red between LA and HP-β-CD. A He–Ne laser with a wavelength of 632.8 nm was used in all of the Raman spectra.

In Figure 1II, SupraDES predominantly exhibits the same bands as LA, mixed with bands at 1081 cm^–1^ (C–O) and 1026 cm^–1^ (C–C) from HP-β-CD. However, the band at 1152 cm^–1^ (C–O–C) appears in SupraDES as a shoulder shifted to 1129 cm^–1^. Furthermore, the band of LA at 1248 cm^–1^, corresponding to −CH and −OH torsion, shifts to 1238 cm^–1^ when forming SupraDES.^53,54^

In the Raman spectrum of SupraDES in Figure 1III, mostly the same bands present in the LA spectrum are observed. However, LA present in SupraDES, when interacting with HP-β-CD, enhances the signal intensity of the band at 770 cm^–1^, corresponding to the vibrational mode of Ca–Cb carbons (Figure 1V). Moreover, in Figure 1IV­[A], it was observed that the band at 894 cm^–1^, corresponding to the Cd–Ce stretching of LA, remains in the same region when present in SupraDES (Figure 1IV­[B]).^55^ For HP-β-CD (Figure 1IV­[C]), the bands at 854 cm^–1^ (O–C3–H) and 950 cm^–1^ (C1–O–C4) remain in the same region as that of the SupraDES spectrum.

However, the band at 930 cm^–1^ corresponding to the C1–O–C2 vibrational mode was shifted to 921 cm^–1^ upon SupraDES formation, indicating interaction with LA,^56^ as shown in infrared (Figure 1II). The interactions described in the text are illustrated by the table and red circles in the diagram in Figure 1V, depicting the interaction of hydroxyl groups and C1–O–C2 of HP-β-CD with the carboxyl region of LA.

### 3.2 Thermal Properties

Dynamic thermogravimetric analysis (TGA) was employed to conduct a detailed investigation of the thermal stability of the SupraDES and to compare it with the thermal analysis of its isolated constituents.

As illustrated in Figure 2, SupraDES underwent progressive decomposition as the temperature increased. The analysis revealed that this decomposition occurred gradually with increasing temperature, displaying a two-stage mass loss pattern similar to choline-based deep eutectic solvents (DES) as described by Delgado-Mellado et al.^57^ The initial decomposition began around 130 °C, and this mass loss was attributed to the degradation of LA.^27^ When examining the thermogram for LA, it is noted that the initial decomposition coincides with that observed in SupraDES. Although stability can vary in mixtures, such as with cyclodextrins, it is interesting to note that the initial decomposition of SupraDES still centers around 130 °C.

**Figure 2:**
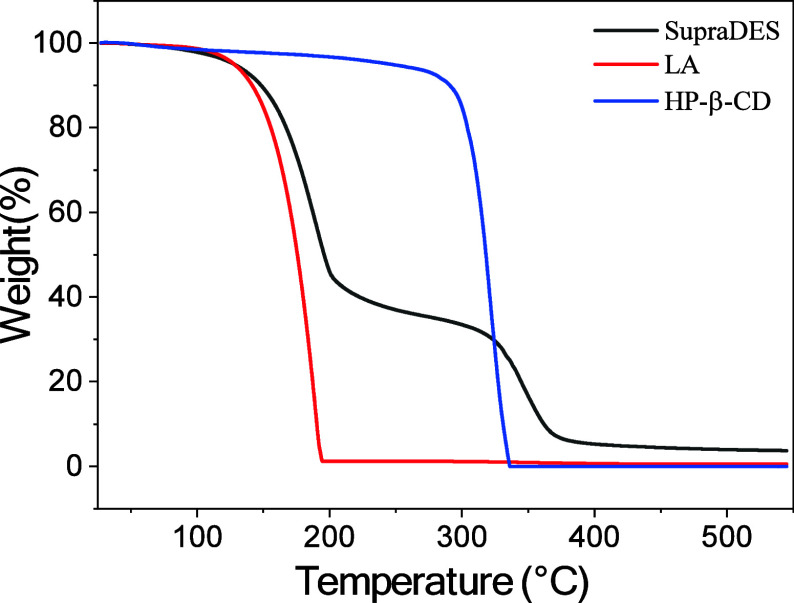
Thermogravimetric analysis of SupraDES and its respective components LA and HP-β-CD.

The second stage of SupraDES decomposition begins around 300 °C corresponding to the decomposition of HP-β-CD. However, when compared with the decomposition of isolated HP-β-CD, the onset of its decomposition is observed at approximately 243 °C, which is lower than the second stage of SupraDES. According to the literature, these compounds exhibit relatively broad melting ranges from 220 to 257 °C.^58^ This shift in the second decomposition point is significant and suggests the occurrence of LA-HP-β-CD interactions, which contribute to the increased stability of the formed compound compared to that of its isolated constituents.

The TGA results demonstrate that SupraDES, based on LA and HP-β-CD, maintains a stable liquid state over a considerable temperature range.

### 3.3 Evaluation of the Effects of Temperature, Time, Amount of SupraDES, and Amount of the CX4SO_3_H Organocatalyst in the Synthesis of FF from d-Xylose

The first parameter that investigated the synthesis of FF from d-xylose was temperature. To assess its impact on the reaction, other parameters were held constant following the protocols outlined in previous studies by Castro et al.,^19^ with minor changes: 1.0 mol % CX4SO_3_H, MW (150 W), and butyl acetate as the extracting phase, with a mass ratio of 1:4 (d-xylose:SupraDES, 50.0 mg:200.0 mg).

Temperatures ranging from 130 to 160 °C were examined. Temperatures exceeding 160 °C could not be explored due to constraints of the MW reactor; experiments at 170 °C led to the loss of butyl acetate due to the elevated system pressure. As illustrated in Figure 3, a gradual increase in FF yield was observed until reaching 150 °C, yielding 60.6% FF. At 160 °C, no significant improvement in yield was noted, thus maintaining 150 °C as the optimal reaction temperature.

**Figure 3:**
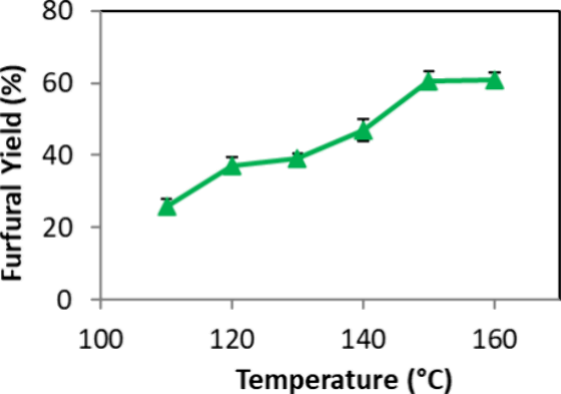
Evaluation of the effect of the temperature of reaction for the synthesis of FF from d-xylose. Reagents and conditions: 50 mg of d-xylose, mass ratio d-xylose/SupraDES (1:4), 1.0 mol % CX4SO_3_H, MW (150 W) and 10 min, and 3.0 mL of butyl acetate.

Subsequently, different reaction times were assessed within the range of 2.5–20 min, with 2.5 min increments. Figure 4 depicts an increase in FF yield with prolonged reaction time, peaking at approximately 10 min with a yield of 60.6%. However, beyond this point, a slight decline in yield was observed. This decline may be attributed to secondary reactions, leading to humin formation, a phenomenon corroborated by the appearance of a dark solid at the reaction’s conclusion, consistent with findings in the prior literature.^15^ Humins arise from the cross polymerization of FF with free d-xylose in solution or reactive intermediates present during the reaction, underscoring the increased likelihood of side reactions with longer reaction times.^59^ Therefore, 10 min was maintained as the optimal reaction time.

**Figure 4:**
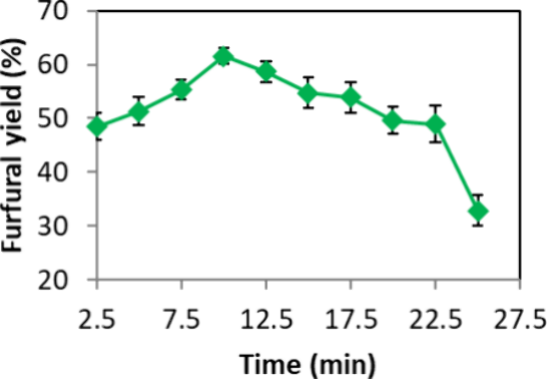
Evaluation of the effect of the reaction time on the synthesis off FF from d-xylose. Reagents and conditions: 50 mg of d-xylose, 1.0 mol % CX4SO_3_H, MW (150 W), 150 °C, and 3.0 mL of butyl acetate.

Subsequently, the effect of the amount of SupraDES used on the FF yield was investigated. To do so, the *m*/*m* ratio of d-xylose/SupraDES was varied from 1:1 to 1:5 as shown in Table 2. The maximum yield (70.6%) was observed at a 1:3 ratio of d-xylose to SupraDES (Table 2, entry 3). However, at ratios of 1:4 and 1:5, a decrease in the FF yield was observed (Table 2, entries 4 and 5). An experiment was also conducted in the absence of SupraDES, revealing a yield of only 26.7% (Table 2, entry 6). Moreover, among the components of the reaction mixture, butyl acetate exhibits a low dielectric constant (∼5.0), which limits its microwave absorption efficiency. In this regard, the role of SupraDES as a heating medium becomes essential. Although HP-β-CD also has a low dielectric constant (∼3.2), its hydroxyl groups and cyclic structure facilitate more effective interactions with the microwave electromagnetic field compared to butyl acetate. Thus, HP-β-CD can function as an “energy transfer facilitator” in microwave-assisted reactions, enhancing the microwave absorption of other substances. Furthermore, LA significantly contributes to selective heating due to its polar groups, which interact with the microwave electromagnetic field, aided by HP-β-CD. Consequently, the observed reaction efficiency (with an FF yield of 70.6%) is likely attributed to the dielectric properties of the SupraDES mixture, which improves microwave absorption and energy transfer.^60–62^

**Table 2:** Study of the *m*/*m* Ratio of d-Xylose/SupraDES in the Yield of FF Production from d-Xylose^a^

**entry**	**mass ratio**	**yield (%)^b^**
1	(1:1)	53.1 ± 1.2
2	(1:2)	64.1 ± 0.6
3	(1:3)	70.6 ± 0.9
4	(1:4)	61.1 ± 0.6
5	(1:5)	62.5 ± 0.8
6	without SupraDES	26.7 ± 0.9

a Reagents and conditions: 50 mg of d-xylose, CX4SO_3_H (1 mol %), 3.0 mL of butyl acetate, MW (150 W), 150 °C, 10 min.

b The yield of FF was calculated from the calibration curve.

The CX4SO_3_H contains acidic groups, such as phenolic groups, which can act as active sites for catalysis.^63^ These acidic groups are capable of protonating xylose molecules, facilitating the removal of a water molecule. This protonation is crucial for dehydration and serves as a key step in the conversion of xylose to furfural.^29^ Castro et al.^15^ proposed a catalytic cycle for the action of CX4SO_3_H during the dehydration of d-xylose to form FF.

Several pathways for the conversion of d-xylose into FF have been reported in the literature, and numerous studies have shown that the reaction route is significantly influenced by the nature of the catalyst employed.^29,64^ In particular, multiple studies have confirmed that FF formation from d-xylose proceeds via a Bro̷nsted acid-catalyzed mechanism, involving a sequence of rearrangements and dehydration steps.^15,64–66^

This same mechanism is likely at play in the present study, which employs a supramolecular deep eutectic solvent (SupraDES) containing LA as a Bro̷nsted acid in combination with the acid catalyst CX4SO_3_H.

As illustrated in Figure 5, the reaction begins with the protonation of xylopyranose at carbon 2 by CX4SO_3_H, followed by dehydration to form a cyclic intermediate. Subsequently, the aldehyde intermediate undergoes protonation at the β-carbon, facilitated by either CX4SO_3_H or LA,^66^ leading to another dehydration step and formation of an α,β-unsaturated aldehyde. A final protonation at carbon 3 triggers the last dehydration, yielding furfural. Beyond acting as a reaction medium, SupraDES stabilizes reactive intermediates and facilitates both protonation and dehydration steps throughout the process.^64^ Overall, this catalytic cycle represents an efficient pathway for converting xylose into FF under mild conditions, offering a promising and sustainable alternative to conventional mineral-acid-based processes for the production of value-added bioproducts.

**Figure 5:**
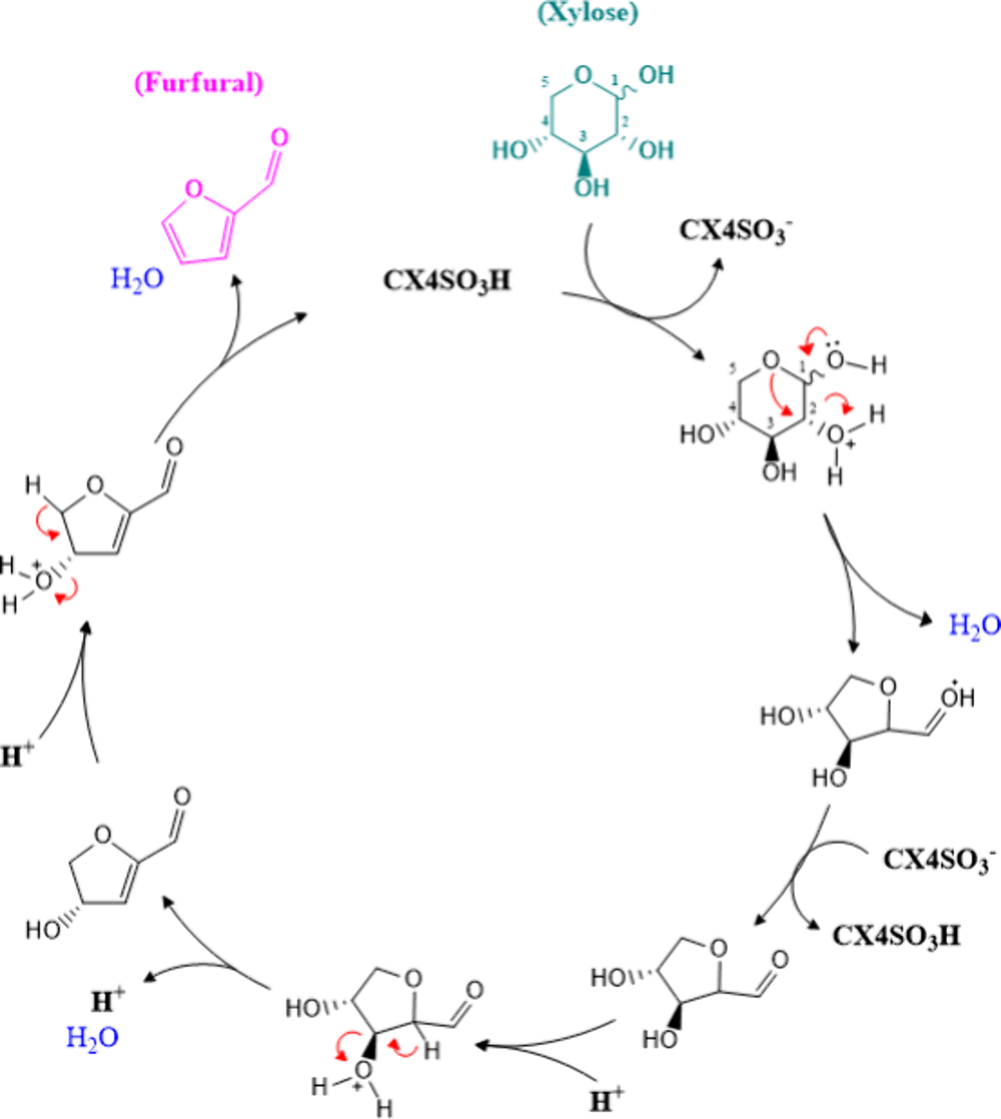
Proposed mechanism for the dehydration reaction of d-xylose to FF.

Figure 6 illustrates the effect of different quantities of the CX4SO_3_H ranging from 0.0 to 2.0 mol %. It was observed that in the absence of the CX4SO_3_H, no FF formation occurred, highlighting the necessity of the organocatalyst for the reaction. With 1.0 mol % CX4SO_3_H, an FF yield of 71% was achieved, indicating an enhancement attributed to the presence of active sites in the solution (Figure 6). However, for quantities exceeding 1 mol %, no significant changes in FF yield were observed; thus, the value of 1.0 mol % CX4SO_3_H was defined as the optimal quantity for the reaction (Figure 6).

**Figure 6:**
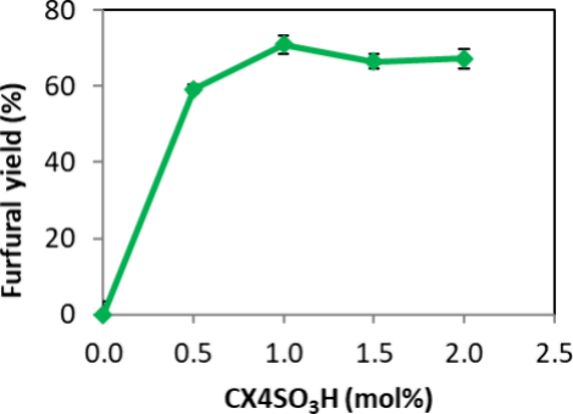
Evaluation of the effect of the CX4SO_3_H amount on the synthesis of FF from d-xylose. Reagents and conditions: 50 mg of d-xylose, MW (150 W), 150 °C, 10 min, and a biphasic system (butyl acetate/mass ratio d-xylose/SupraDES (1:4).

### 3.4 Investigation of the Hydrogen Bond Donor (HBD) Nature in SupraDES Composition on the Synthesis of FF from d-Xylose

The nature of the hydrogen bond donors (HBDs) in the composition of SupraDES was investigated. The following natural organic acids were evaluated: AA, CA, LaA, and LA. Table 3 demonstrates that the acidity of the HBD significantly influences the conversion of d-xylose to FF, as SupraDESs formed by monoprotic acids (Table 3, entries 1, 3, and 4) led to an increase in FF yield with the increase in the acid’s p*K*_a_. However, for CA, it was observed that the acid promoted superior conversion compared to LaA, despite having a lower pKa1 value. A possible explanation may be related to the acids interacting with their carboxyl group with HP-β-CD, as shown by IR and Raman analysis of LA: HP-β-CD, altering its dissociation constant and consequently its acid strength. Similarly, Qin et al. (2020) reported that the DES formed by CA and benzyl trimethylammonium chloride (BTMAC) caused a change in the acid’s pKa1, increasing from 3.13 (tabulated value) to 4.14 when present in the DES.^67^

**Table 3:** Evaluation of Different Natural Organic Acids in SupraDES^a^

entry	organic acid	p*K*_a__1_ ^c^	FF (%)^b^
1	levulinic	4.78	71.0 ± 0.9
2	acetic	4.76	46.6 ± 1.2
3	citric	3.13	36.6 ± 0.6
4	lactic	3.86	27.3 ± 0.6

a Reagents and conditions: CX4SO_3_H (1.0 mol %), 150 mg of SupraDES, 3.0 mL of butyl acetate, MW (150 W), 150 °C, 10 min.

b The yield of FF was calculated from the calibration curve.

c p*K*_a_ constants (in water) tabulated.^68^

### 3.5 Study of the Application on Different Substrates for FF and HMF Synthesis

After determining the best conditions for the synthesis of FF from d-xylose (CX4SO_3_H (1.0 mol %), 150 mg of SupraDES, 3.0 mL of butyl acetate, MW (150 W), 150 °C, 10 min), it was decided to evaluate other substrates for the synthesis of FF and also HMF (Table 4). When evaluating l-arabinose (d-xylose diastereoisomer)^69^ as a substrate for the synthesis of FF, it was observed that the yield obtained was much lower than when d-xylose was used as a substrate (Table 4, entries 1 and 2). It is important to highlight that this difference has already been observed in previous works and that it can be justified by the difference in the activation energies of the dehydration reactions of the two substrates since that of d-xylose is always lower than that of l-arabinose. Therefore, the dehydration reaction of l-arabinose occurs more slowly and requires higher temperatures to speed up the process.^70^ This phenomenon has also been observed in previous studies^71,72^

**Table 4:** Evaluation of Different Substrates for the Synthesis of FF and HMF^a^

entry	substrate	FF (%)^b^	HMF (%)^b^
1	d-xylose	70.6 ± 0.9	
2	l-arabinose	17.6 ± 0.9	
3	d-fructose		28.7 ± 0.7
4	inulin from Dahlia tubers		29.7 ± 0.9
5	d-glucose		0.0 ± 0.0
6	corn cob biomass	1.3 ± 0.8	0.0 ± 0.0
7^c^	corn cob biomass	2.3 ± 0.6	0.0 ± 0.0

a Reagents and conditions: CX4SO_3_H (1.0 mol %), 150 mg of SupraDES, 3.0 mL of butyl acetate, MW (150 W), 150 °C, 10 min.

b The yields of FF and HMF were calculated from the calibration curve.

c An experiment with a time of 60 min.

Substrates for HMF synthesis were also evaluated: d-fructose, inulin (d-fructose polymer, obtained from Dahlia tubers), and d-glucose (Table 4, entries 3–5). For d-fructose and inulin, HMF was obtained with yields of 28.7 and 29.7%, respectively (Table 4, entries 3 and 4). The formation of humins was observed in these experiments, which leads to lower yields of HMF due to the occurrence of parallel reactions that consume the product and substrate.^17,18,29,31^ For d-glucose, the formation of HMF was not observed (Table 4, entry 5). This occurred because the transformation of d-glucose into HMF follows a two-step process, in which d-glucose is first isomerized into fructose, followed by subsequent dehydration of fructose into HMF. For this process to be efficient in converting d-glucose into HMF, it is required the combined use of a Lewis acid catalyst, for the isomerization of d-glucose into d-fructose, and a Bro̷nsted acid catalyst, to promote the dehydration of d-fructose into HMF.^42–44^ Therefore, as the system presented here has only Bro̷nsted acid-type catalysts, the formation of HMF from d-glucose is not observed (Table 4, entry 5).

Finally, corncob biomass was also evaluated for the synthesis of FF and HMF, (Table 4, entry 6), as it is rich in cellulose and hemicellulose, polysaccharides consisting mainly of d-glucose and d-xylose, respectively.^45,46^ However, FF was obtained in low yield, even when employing a reaction time six times longer (Table 4, entry 7). This probably occurred because for a reaction that uses biomass as a substrate, the depolymerization of the hemicellulose chains and the release of d-xylose and l-arabinose must occur.^29,41^ These monosaccharides undergo dehydration reactions that lead to the formation of FF. Therefore, the system was possibly not efficient in depolymerizing the hemicellulose chains. Furthermore, the formation of HMF was also not observed (Table 4, entry 7), which is justifiable given that the main carbohydrate that produces HMF found in lignocellulosic biomass is d-glucose. Also, the system was not able to produce HMF even from isolated d-glucose (Table 4, entry 5).

The FF produced by the dehydration of d-xylose using CX4SO_3_H as an organocatalyst and SupraDES in a biphasic system achieved yields comparable to or higher than those reported in previous studies. Zhang and Yu^73^ employed monophase (DES) and biphasic (DES/MIBK) systems with trivalent metal chlorides but obtained moderate yields (14–44%), with the maximum values of 60.4% for xylose and 55.5% for xylan. Additionally, the system has thermal limitations, with instability at temperatures above 100 °C, restricting its industrial applicability. The use of metal chlorides also undermines process sustainability due to the environmental impact and difficulties in catalyst recycling. Wang et al.^100^ achieved up to 70.3% yield using DESs with Lewis and Bro̷nsted acids, but with significantly long reaction times (90 min at 140 °C) and the need for MIBK, which presents challenges with phase miscibility and separation, as well as being toxic and expensive.

Delgadillo et al.^29^ developed a green methodology for the synthesis of furfural FF using a biphasic system. In this system, the reaction phase consisted of the deep eutectic solvent DES made from ChCl, water, PTSA, and d-xylose, while the extraction phase used ethyl acetate. Using this setup, FF was obtained with a yield of 50% by heating the mixture at 130 °C for 15 min in a microwave reactor. Li et al.^74^ investigated the feasibility of chemoenzymatically catalyzing the conversion of d-xylose into furfuryl alcohol through tandem catalysis with HCO_2_H and reductase in a deep eutectic solvent (DES)–water medium. An FF yield of 58.3% was achieved by catalyzing biomass-derived d-xylose (60.0 g/L) for 30 min at 180 °C with HCO_2_H (3.0 wt %) as the catalyst, in the presence of DES ChCl:glycose (7.5 vol %).

On the other hand, this study offers a more efficient and sustainable process, with higher yields and fewer operational complexities. The proposed methodology stands out not only for its high yield and mild operating conditions but also due to the absence of metals in the process, the use of an environmentally friendly and low-toxicity organocatalyst, and its demonstrated economic feasibility.

### 3.6 Techno-Economic Assessment (TEA)

The production capacity for this process was set at 1 ton of FF per day, operating 350 days annually, leading to an annual capacity of 350 tons (Figure S4). A comprehensive mass balance determined that the global yield was approximately 11%, requiring 9.52 tons of bamboo biomass per ton of FF. Yield metrics for the intermediate steps were also established: the acidic hydrolysis of bamboo biomass achieved a 15% yield of d-xylose, 3.33 tons of bamboo are need to extract 1.00 tons of lignin,^75^ and the catalytic conversion of d-xylose to FF yielded 70% (Table 5).

**Table 5:** Key Assumptions for the Techno-Economic Assessment of Furfural (FF) Production from Bamboo Biomass, Including Yields, Production Capacity, and Financial Parameters

category	value
private capital financing (%)	10
availability (days/y)	350
depreciation (y)	25
opportunity cost SELIC + 3% (%)	10
production capacity (tons/day)	1
production capacity (tons/y)	350
total annual operating hours (h)	8400
total CAPEX (USD)	22,500.00
bamboo acid hydrolysis yield—d-xylose (%)	15
d-xylose to furfural yield (%)^a^	70.6
overall yield (t bamboo biomass/t FF)	10.59
lignin yield (t bamboo biomass/t Lignin)	3.3

a According to the results obtained in this study. To estimate operational expenditures (OPEX), specific costs for each input were calculatedbased on industry pricing.

This venture was designed to benefit from significant financial support through government agency incentives, with 90% funding anticipated as part of environmental policy programs. This strategic funding approach aligns with governmental efforts to promote sustainable industrial practices, enabling a more feasible and scalable implementation of the production process.

### 3.7 TEA Main Indicators and Annual OPEX

The economic evaluation revealed that the production of FF from bamboo biomass has a considerable financial viability. The cash operating cost was calculated at USD 8107.81 per ton of FF, driven primarily by the costs of bamboo biomass, catalyst, HP-β-CD, and LA. Bamboo biomass, the primary feedstock, was consumed at a rate of 9.52 tons per ton of FF, with an annual demand of 3333 tons. The cost for the catalyst, at USD 67.00 per kg, added USD 4422.00 per ton of FF, while HP-β-CD and LA contributed an additional USD 1500.00 and USD 2129.34 per ton, respectively (Table 6).

**Table 6:** Annual Operational Expenditures (OPEX) for Furfural Production, Detailing Costs for Raw Materials, Catalysts, Solvents, and Utilities

category	value	reference
depreciation cost (USD/y)	900.00	^a^
depreciation cash cost (USD/t FF)	2.57	^a^
specific bamboo consumption (t bms/t FF)	9.52	^a^
bamboo price (USD/t bms)	5.29	^76^
annual bamboo consumption (t bms/y)	3333.33	^a^
bamboo cash cost (USD/t FF)	50.38	^a^
catalyst CX4SO_3_H quantity (kg/t FF @1.0 mol %)	66.00	^a^
catalyst CX4SO_3_H price (USD/kg)	67.00	^77^
catalyst CX4SO_3_H cash cost (USD/tFF)	4422.00	^a^
butyl acetate quantity (kg/t FF)	0.0000800	^a^
butyl acetate price (USD/kg)	1.03	^78^
butyl acetate quantity cash cost (USD/t FF)	0.0000001	^a^
HP-β-CD quantity (kg/t FF)	1200.00	^a^
HP-β-CD Price (USD/kg)	1.25	^79^
HP-β-CD cash cost (USD/t FF)	1500.00	^a^
LA quantity (kg/t furfural)	3086.00	^a^
LA price (USD/kg)	0.69	^80^
LA cash cost (USD/t FF)	2129.34	^a^
specific H_2_SO_4_ consumption (kg/t FF)	42.86	^a^
H_2_SO_4_ market price (USD/t)	5.68	^81^
H_2_SO_4_ cash cost (USD/t FF)	0.24	^a^
specific electric energy consumption (MWh/t FF)	0.15	^a^
electric energy market price (USD/MWh)	21.82	^82^
electric energy cash cost (USD/tFF)	3.27	^a^
total cash cost (fixed + variable) (USD/t FF)	8107.81	^a^
annual OPEX	2,837,732.99	^a^

a Values are estimated based on experimental data and authors’ expertise.

At a market price of USD 6445.64 per ton,^83^ FF production generated an annual revenue of USD 2,255,974.00. Additionally, lignin, a valuable byproduct, was produced at a rate of 1000 tons annually and sold at USD 750.00 per ton,^84^ adding USD 750,000.00 in revenue. Consequently, the total revenue from FF and lignin reached USD 3,005,974.00 per year (Table 7).

**Table 7:** Gross Operating Revenue (GOR) Projections from Furfural and Lignin Sales, Including Production Volumes and Market Prices

category	value
FF production (t/y)	350
FF price (USD/t)	6445.64
GOR (USD/y FF)	2,255,974.00
lignin production (t/y)	1000
lignin price (USD/t)	750.00
GOR (USD/y lignin)	750,000.00
total GOR (USD/y)	3,005,974.00

Key financial indicators demonstrated strong economic performance with a payback period of just 0.13 years, an IRR of 75%, and an ROI of 65%. The NPV was calculated at USD 1,183,754.91, with a gross margin of 6%, underscoring the high profitability and quick capital recovery of this production method. These results suggest that bamboo-based FF production, supported by an innovative catalytic process, is economically feasible and competitive in the renewable chemical market.

For future research, efforts should focus on SupraDES charge optimization to potentially enhance the cost efficiency and catalytic effectiveness. Additionally, exploring other organic-based solvents could yield valuable comparisons, helping to identify alternative solutions that may improve sustainability and economic viability. Such further investigations could enhance the process, potentially boosting yields or lowering overall production costs, thus contributing to the advancement of greener industrial practices.

## 4 Conclusions

An efficient method was developed for synthesizing FF and HMF from model compounds derived from lignocellulosic biomass assisted by microwave irradiation (MI). The process uses a SupraDES (HP-β-CD), the organocatalyst CX4SO_3_H, and butyl acetate as an extractor. A yield of 70.6% ± 0.9 of FF was achieved at 150 °C for 10 min with 1 mol % CX4SO_3_H using MW, starting from d-xylose. A techno-economic assessment was conducted for FF production via bamboo hydrolysis. The financial results demonstrated robust economic viability, with a payback period of 0.13 years, an IRR of 75%, an ROI of 65%, and an NPV of USD 1,183,754.91.

The CX4SO_3_H catalyst, as a nontoxic and chemically stable organocatalyst, plays a key role in the reaction by efficiently promoting the dehydration of xylose in an environmentally friendly manner. The two-phase system, which contains a SupraDES formed by the combination of a biomass-derived platform molecule LA and a sugar (HP-β-CD), operates in a metal-free process. Both HP-β-CD and LA are renewable, green, and nontoxic sources, making this system even more environmentally attractive. These conditions prove to be advantageous and well-suited for industrial applications, offering a new perspective on the potential of SupraDESs as solvents for converting carbohydrates to chemical platforms.

## Supplementary Material



## Notes

Chromatograms, mass spectra, and process flow diagram (PDF)

## Abbreviations

FF: furfural
HMF: 5-hydroxymethylfurfural
CX4SO_3_H: *p*-sulfonic acid calix[4]­arene
HBD: hydrogen bond donors
HDA: hydrogen bond acceptor
DES: deep eutectic solvents
SupraDES: deep solvent eutetic supramolecular
AA: acetic acid
CA: citric acid
LaA: lactic acid
LA: levulinic acid
HP-β-CD: 2-hidroxipropyl-β-cyclodextrin
MI: microwave irradiation
y: year
TEA: techno-economic assessment
OPEX: estimate operational expenditures
CAPEX: capital expenditures
IRR: internal rate of return
ROI: return on investment
GOR: gross operating revenue
