# Supplementary material for: Green Synthesis of Furfural and Hydroxymethylfurfural from Various Substrates Using SupraDES under Microwave Irradiation: Techno-Economic Evaluation
Source: ACS Omega. 2025 Jun 4;10(23):24576–86. doi: 10.1021/acsomega.5c01297 (PMC12177752; doi:10.1021/acsomega.5c01297)
Supplement: Supplementary file 1 [file ao5c01297_si_001.pdf]

Green synthesis of furfural and hydroxymethylfurfural from various substrates using SupraDES under microwave irradiation: Techno-economic evaluation

Lorena Cristina de Andrade Leles[a], *Gabriel Abranches Dias Castro*[a], *Jaderson Lopes Milagres*[b], *Juliana Ribeiro Paes*[a], *Leonarde do Nascimento Rodrigues*[c], *Ricardo de Carvalho Bittencourt*[d], *Marcelo Moreira da Costa*[d], *Sergio Antonio Fernandes*[*a]

[a]Grupo de Química Supramolecular e Biomimética (GQSB), Departamento de Química, Universidade Federal de Viçosa, Viçosa, MG, 36570-900, Brazil

[b] Departamento de Ciências Exatas e da Terra, Universidade do Estado de Minas Gerais, Ubá, MG, 36500-000, Brazil

[c] Departamento de Física, Centro de Ciências Exatas, Universidade Federal de Viçosa, Viçosa, Minas Gerais, 36570-900, Brazil

[d] Departamento de Engenharia Florestal, Universidade Federal de Viçosa, Viçosa, MG ,36570-900, Brazil

[*]e-mail: santonio@ufv.br or sefernandes@gmail.com

Quantification and Identification of FF and HMF

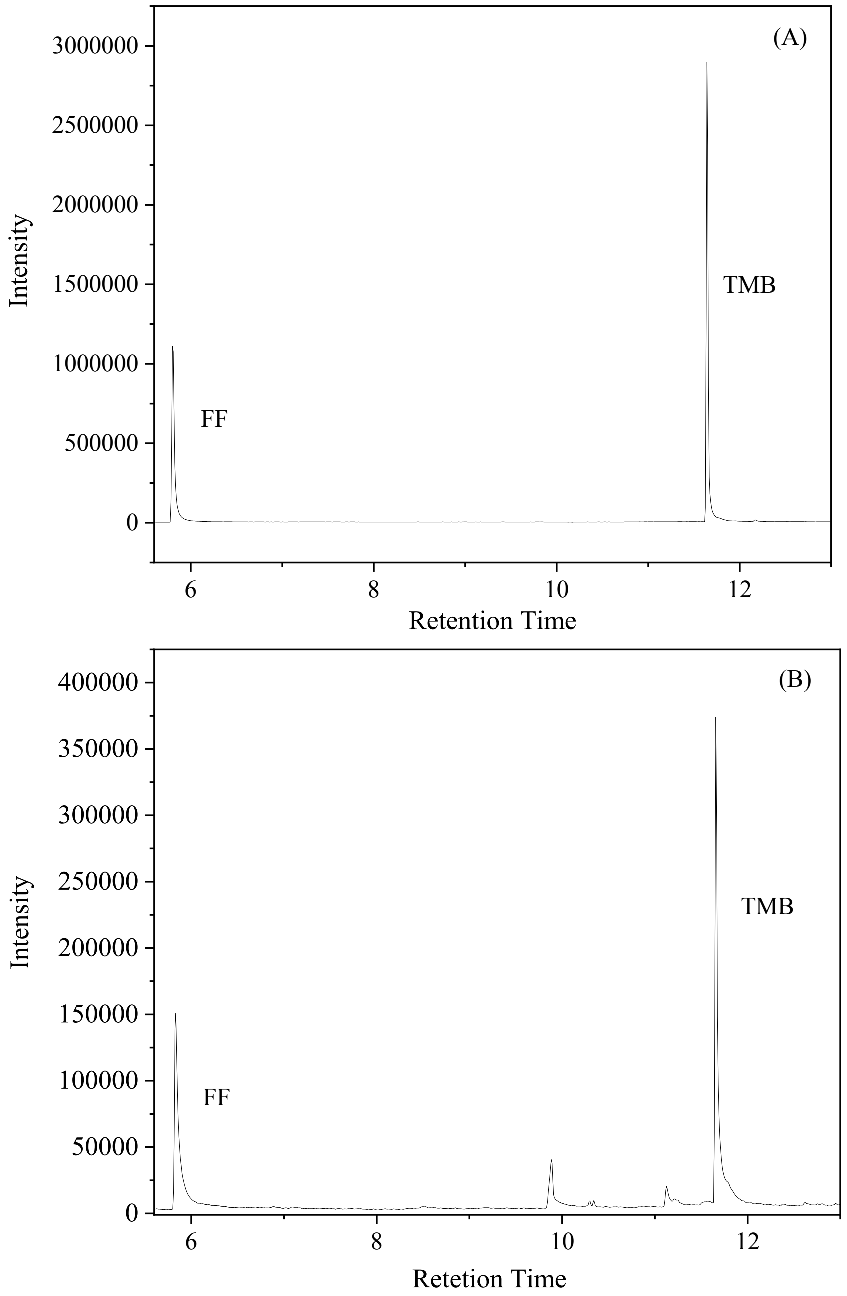

Fig. S1. (A) Chromatogram of the furfural (FF) standard at a concentration of 1.00 mg/mL and 1,3,5-trimethoxybenzene (TMB) at 1.00 mg/mL. (B) Chromatogram showing the synthesis of furfural (FF) from *D*-xylose.

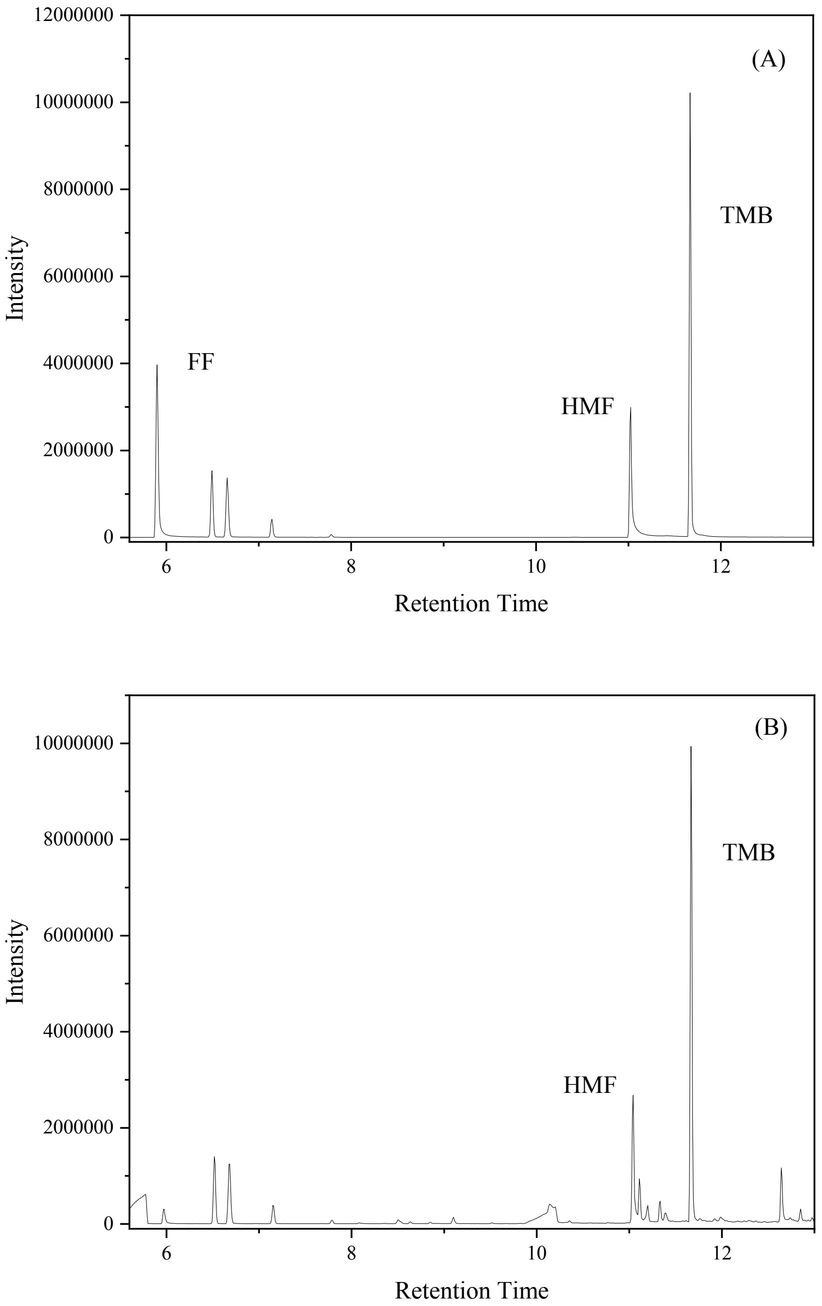

Fig. S2. (A) Chromatogram of the furfural (FF) and 5-hydroxymethylfurfural (HMF) standards at a concentration of 1.00 mg/mL each, and 1,3,5-trimethoxybenzene (TMB) at 1.00 mg/mL. (B) Chromatogram of the synthesis of furfural (FF) and 5-hydroxymethylfurfural (HMF) from *D*-fructose.

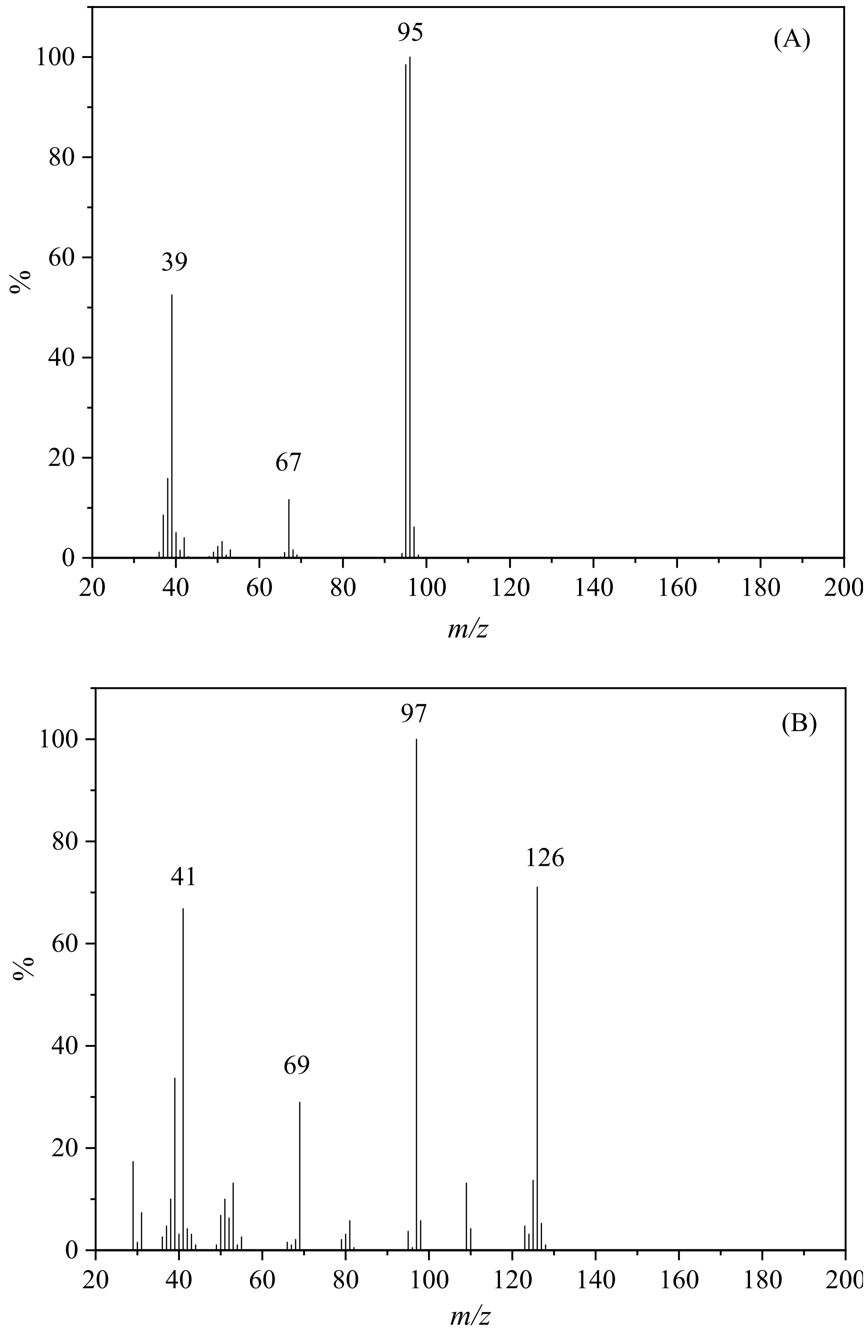

Fig. S3. (A) Mass spectrum of furfural (FF); (B) Mass spectrum of 5-hydroxymethylfurfural (HMF).

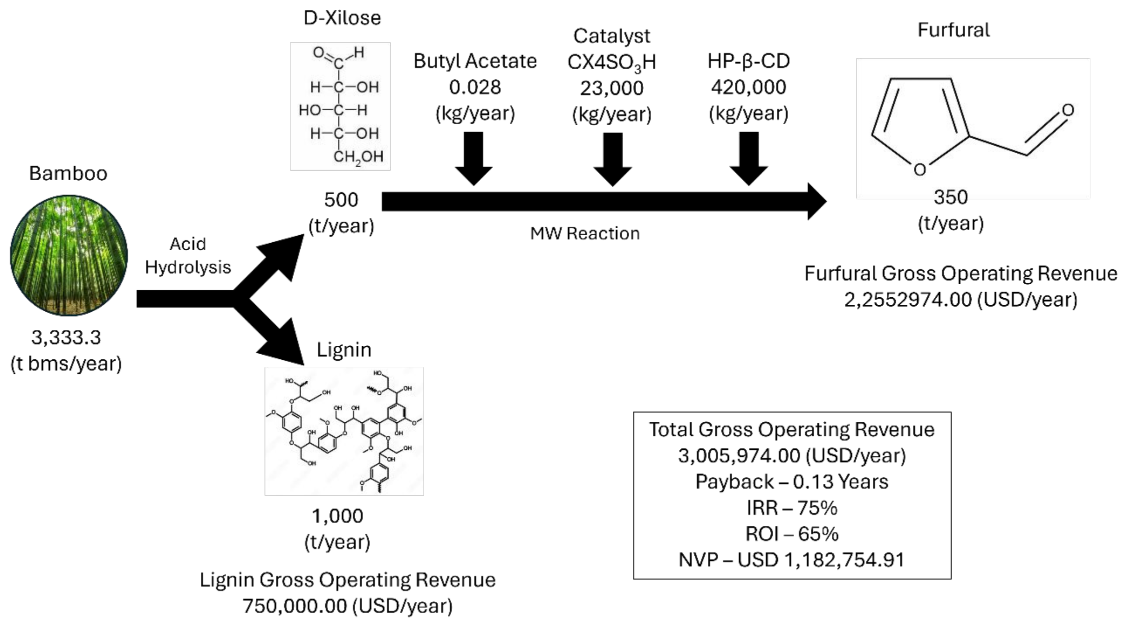

Fig. S4. Process flow diagram of the entire production process evaluated in the Techno-Economic Assessment (TEA), including key conversion steps, inputs, products, by-products, and energy flows.
